# The Epidermis in Microgravity and Unloading Conditions and Their Effects on Wound Healing

**DOI:** 10.3389/fbioe.2022.666434

**Published:** 2022-03-22

**Authors:** Stefano Bacci, Daniele Bani

**Affiliations:** ^1^ Research Unit of Histology and Embryology, Florence, Italy; ^2^ Department Biology, Florence, Italy; ^3^ Department, Experimental and Clinical Medicine, University of Florence, Florence, Italy

**Keywords:** microgravity, keratinocytes, epidermis, epidermal stem cells, skin, wound healing

## Abstract

The future objectives of human space flight are changing from low-term permanence in the International Space Station to missions beyond low Earth orbit to explore other planets. This implies that astronauts would remain exposed for long time to a micro-gravity environment with limited medical support available. This has sparkled medical research to investigate how tissues may adapt to such conditions and how wound repair may be influenced. This mini-review is focused on the effects of microgravity and unloading conditions on the epidermis and its keratinocytes. Previous studies, originally aimed at improving the *in vitro* protocols to generate skin substitutes for plastic surgery purposes, showed that epidermal stem cells cultured in simulated microgravity underwent enhanced proliferation and viability and reduced terminal differentiation than under normal gravity. In the meantime, microgravity also triggered epithelial-mesenchymal transition of keratinocytes, promoting a migratory behavior. The molecular mechanisms, only partially understood, involve mechano-trasduction signals and pathways whereby specific target genes are activated, i.e., those presiding to circadian rhythms, migration, and immune suppression, or inhibited, i.e., those involved in stress responses. However, despite the above *in vitro* studies suggest that microgravity would accelerate keratinocyte growth rate and migration, *in vivo* findings on animals in experimental set-ups to simulate low gravity rather suggest that prolonged mechanical unloading contributes to delayed and impaired epidermal repair. This is in keeping with the finding that microgravity interferes at multiple levels with the regulatory signals which coordinate the different cell types involved in the repair process, thereby negatively influencing skin wound healing.

## Introduction

The new millennium has initiated a new chapter of space exploration. The many data gathered upon the unmanned missions to Mars have supported the feasibility of long-range space flights to bring astronauts to explore the closer planets, a perspective that may become something more than a screenplay for a science-fiction movie. To make this possible, many scientific issues have to be considered and managed, especially those related to the physical and mental health of crews exposed for unprecedented long times to the confined environment of a spaceship under low gravity conditions, and limited availability of medical resources. Studies on astronauts who performed enduring missions in the International Space Station (ISS) have allowed to collect numerous medical data on the effects of low gravity on the human body, crucial to identify microgravity-induced diseases (Sekiguchi, 1994; Smith et al., 2014; Richter et al., 2017), as well as their pathogenic mechanisms at the cellular and molecular level (Pietsch et al., 2011). The best-known adverse effects of low gravity exposure, yet emerged upon short-term low-Earth-orbit permanence, consist in bone and muscle loss, reduction of cardiovascular capacity, delayed wound and bone fracture healing, and impaired immune function. Over the long-term, exposure to microgravity may impair stem cell-dependent tissue regeneration and homeostasis, adversely affecting bone formation and remodeling, and hemato/lymphopoiesis. In this context, long-term low gravity experiments on amphibians have demonstrated the loss of ability of stem cells of blastemata to regenerate the tail and ocular lens (Bizzarri et al., 2015). Another reason for concern is the susceptibility of astronauts to trauma due to peculiar working needs and conditions, since observational data suggest an impaired response to wounding and injury, such as the unusual behavior of hemorrhage, microbiologic flora, and wound healing (Kirkpatrick et al., 1997). This has sparkled medical research to investigate how tissues, particularly the skin, may adapt to such conditions, and how wound repair may be influenced. This mini-review will be focused on the effects of microgravity and unloading conditions on the epidermis and its keratinocytes, viewed in the context of their contribution to the wound healing process of the skin.

## Keratinocytes, Stem Cells and Epidermal Homeostasis

Keratinocytes are directly involved in several functions of the epidermis relevant for healing of skin wounds: first, they proliferate to maintain epidermal tissue homeostasis and repair tissue losses; second, they produce an array of cytokines and growth factors involved in the autocrine regulation of keratinocyte proliferation, migration, and differentiation, as well as in paracrine effects on stromal, inflammatory and immune cells (Yang et al., 2020). Epidermal cell homeostasis results from the continuing activity of the so-called epidermal proliferative unit (EPU), which encompasses undifferentiated epidermal stem cells (ESCs), transit amplifying (TA) cells and committed keratinocytes which, in normal conditions, evolve in terminally differentiated corneocytes within 20–30 days. Approximately, a single ESC yields by asymmetric mitosis 2 siblings, another ESC and a rapidly dividing TA from which approx. 32 terminal keratinocytes arise (Jones, 1997). Typically, ESCs are mainly harbored in stem cell niches located in hair follicle bulges, from which they can settle in the basal layers of interfollicular epidermis and sebaceous glands (Jones, 1997). Interestingly, evidence has emerged that ESCs from the different sites follow their own differentiation paths in normal homeostasis of the epidermis and its annexes, whereas all of them can synergize to give rise to any differentiated epidermal/annexal cell type in response to skin injury (Ito et al., 2005; Watt & Jensen, 2009). Obviously, these populations of ESCs and TA keratinocytes are the most susceptible to regulatory signals, including micro-mechanical stimuli: hence, their disruption in altered gravity conditions can have an impact on homeostasis of the epidermis and its ability to respond to injuries.

## Keratinocytes and Microgravity: Effects and Possible Mechanisms

How microgravity influences the morpho-functional features of keratinocytes and their contribution to skin wound healing is a poorly explored field. Most cell types are able to respond to mechanical cues that activate specific sensor molecules, chiefly integrin-extracellular matrix (ECM) pairings, intercellular adhesion molecules and junctions, ion channels, α/βcatenins, and cytoskeletal components which transduce them into molecular signals modulating cell morphology, proliferation, differentiation and migration (Janmey and McCulloch, 2007; Farahani and DiPietro, 2008). Therefore, it is conceivable that abnormal micro-mechanical stimuli, as those operating in microgravity, may have an impact on keratinocyte behavior, especially when resting cells are aroused to the dynamic condition needed for wound healing.

Previous *in vitro* studies, originally aimed at improving the *in vitro* protocols to generate skin substitutes for plastic surgery purposes, showed that human ESCs cultured in simulated microgravity underwent enhanced proliferation and viability and reduced terminal differentiation as compared with those cultured under normal gravity condition, albeit they retained the capability to form a multi-layered epidermis-like tissue (Lei et al., 2011). Later investigations with human immortalized keratinocytes exposed for up to 60 h to simulated microgravity by a random-positioning machine have revealed changes in the expression and periodicity of *Bmal1* gene involved in the regulation of circadian rhythm, not accompanied by substantial changes in overall cell morphology, proliferation and apoptosis rates, and at least at the explored experimental times (Ranieri et al., 2015). Concurrently, exposure of human keratinocytes to the same simulated microgravity conditions was shown to trigger epithelial-to-mesenchymal transition (EMT), mediated by over-expression of specific transcription factors and markers, such as Snail1, Snail2, *ZEB2*, MMPs, and ECM adhesion molecules, as well as by re-arrangement of cytoskeletal components in a pro-motile pattern (Ranieri et al., 2017). As will be discussed in more detail in a following section, EMT is a first key step required to promote a migratory behavior of keratinocytes whereby they accelerate re-epithelization during wound healing.

The molecular signals and mechanisms involved in these cellular effects of microgravity are only partially understood. Since epidermal growth factor (EGF) and other molecules of the EGF family, which also include TGF-α and heparin-binding EGF, are major regulators of epithelial cell growth and differentiation and are known to play a pivotal role in re-epithelization during the early steps of wound healing (Steed, 1998) it appeared conceivable that these could be altered under low gravity conditions. Indeed, exposure of a human squamous cell line to low gravity was shown to decrease the expression of key genes (*c-fos*, *c-jun*) downstream EGF activation involved in cell cycle progression, likely through an interference with protein kinase C-dependent signal transduction and actin cytoskeleton (Rijken et al., 1994; Boonstra, 1999).

In view of a possible translation of the results of *in vitro* microgravity experiments to space medicine, studies were performed to ascertain whether the microgravity-induced changes of keratinocytes were reversible upon restoration of normal gravity. Of note, human keratinocytes are capable to recover a static epithelial phenotype, as assessed by re-establishment of intercellular adherent junctions and normal cytoskeletal features, mirrored by reduction of their mesenchyme-like migratory phenotype (Ricci et al., 2021). On the other hand, microarray studies on gene expression patterns by human keratinocytes exposed to microgravity for up to 10 days have shown that the longer the exposure to low gravity, the slower and less complete the return to a normal cell morphology and gene expression profile (Clement et al., 2008), In partial agreement with these findings, recent data from cultured endothelial cells suggested that short-term microgravity post-transcriptionally modulated the expression of several genes involved in angiogenesis and vascular patterning (Kasiviswanathan et al., 2020). Other studies have shown that cells in cultures recovered from spaceflight did not migrate normally, as occurs during epithelial wound closure (Almeida, 2011) and that their cytokine, and growth factor secretion pattern is altered (Huang et al., 2020). These results suggest that the migratory and paracrine ability of microgravity-exposed precursor cells is impaired and support the hypothesis that the tissue regenerative potential of stem cells, including ESCs, and may be decreased during spaceflight (Blaber et al., 2014). The positive aspect is that chromosomal, DNA damage, and tumorigenicity assays performed upon return of cell cultures to Earth showed no signs of damage which could be related to malignant transformation (Huang et al., 2020).

A major limitation of the above findings consists in the fact that they have been obtained by *in vitro* cell culture experiments, in which cells are distanced from the complex network of signals they would receive in their physiological tissue environment. This is especially true for skin wound healing, characterized by a functional co-ordination between different player cells which, besides epidermal keratinocytes, also include platelets, inflammatory cells, mesenchymal stem cells, fibroblasts, and endothelial cells (Martin and Nunan, 2015; Dekoninck and Blanpain, 2019). Nonetheless, the above *in vitro* data can rise concerns about the adverse effects of long-lasting space missions on epidermal integrity. Additional background to concerns was provided by *in vivo* studies on skin wound healing using the tail-suspended hindlimb-unloaded rat model to induce functional disuse and intended to simulate microgravity. These studies demonstrated that keratinocyte migration and wound closure were delayed in the mechanically unloaded rats. These effects were accompanied by lower density of dermal microvessels, which also lacked the directional growth toward the epidermis typical of normal skin (Radek et al., 2008). These results collectively indicate that both keratinocyte and endothelial cell function are impaired during wound healing in unloading conditions, likely because of alterations of the complex interplay of regulatory signals exchanged between the epidermis and dermis in the skin as a whole. However, due to the extreme complexity in tuning up reliable microgravity animal models, not to mention true spaceflight *in vivo* experiments (Globus and Morey-Holton, 2016), the scientific data required to better understand how epidermal cells behave during healing of skin wounds in low gravity and the exact mechanisms involved are limited yet.

## Wound Healing: The Roles of Keratinocytes and the Effects of Microgravity

Wound healing is the process that makes organisms resilient to injuries, allowing survival. Being of fundamental importance for life, it basic pathways and mechanisms have been substantially conserved throughout evolution (Eming et al., 2014), although the final outcome diverges from lower vertebrates—like fishes and amphibians—which retain throughout life the embryonic capability to regenerate the missing tissues and organs, and upper vertebrates, which rather heal by reparative scarring (Odelberg, 2005; Bani and Nistri, 2014). As mentioned above, wound healing results from an interplay of diverse cells involved and is classically divided into three phases: inflammation, proliferation, and remodeling, whose mechanisms are partially overlapped both spatially and temporally. Detailed analysis of the complex events and molecular mechanisms of wound healing is outside the scope of this mini-review; here, we will limit to recapitulate the key points. After an injury, clotting suddenly takes place due to immediate interactions among endothelial cells and platelets and activation of the coagulation cascade. Mediators released during this early process trigger an inflammatory reaction summoning neutrophils and macrophages from the bloodstream: in turn, these cells produce pro-inflammatory cytokines and growth factors, resulting in recruitment of stromal cells and their differentiation into myofibroblasts, which are responsible for wound contraction and ECM deposition, and stimulation of endothelial and epithelial cell proliferation in the wound site to induce neoangiogenesis and re-epithelialization, respectively. Clot and tissue debris are eventually removed by macrophages and extracellular hydrolases (matrix metalloproteases MMPs, elastase and plasmin) and tissue repair proceeds towards and terminates with scarring (Singer and Clark, 1999; Li and Kirsner, 2005; Eming et al., 2014; Martin and Nunan, 2015; Thiruvoth et al., 2015).

Many factors can impair wound healing. A crucial one is tissue ischemia, which may be caused by primary vascular diseases, diabetes and persistent local pressure. Another adverse factor is persistence of inflammation, as occurs in necrotic and chronically infected wounds and in burns, which leads to inactivation of growth factors and other molecular stimuli required for tissue repair, trapped by ECM molecules or degraded by extracellular proteases (Han and Ceiley, 2017). A further factor is persistence of myofibroblast activation, leading to hypertrophic scars, or keloids (Martin and Nunan, 2015; Berman et al., 2017). Complicated wound healing represent a major public health issue, as it requires complex and lengthy treatments, prolonged hospitalization and an increasing burden on healthcare expenses. These problems become even more challenging when transposed to space medicine, considering the limited therapeutic options available to astronauts during long-lasting space flights (Cialdai and Monici, 2013).

**FIGURE 1:**
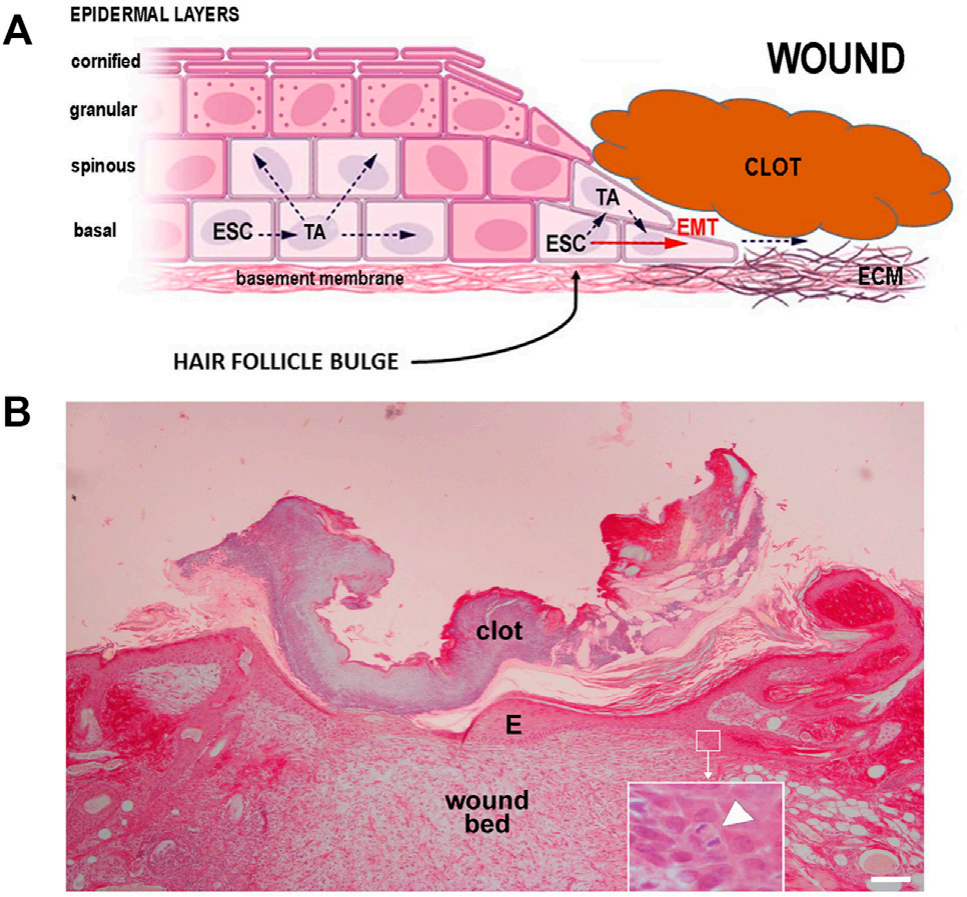
**(A)** Schematic diagram of epidermal keratinocyte proliferation and migration during wound closure: ESC, epidermal stem cells; TA transit amplifying cells; EMT, epithelial-mesenchymal transition; ECM, extracellular matrix. **(B)** Wound healing in rat skin: a thin, flattened layer of newly formed epidermis (E) lies over the wound bed and under the clot; the inset shows a detail of an ESC undergoing mitosis (arrowhead). Hematoxylin, picrosirius red, scale bar = 100 μm (courtesy Dr. P. Nardini).

Keratinocytes play a major role in wound healing since they are activated during the inflammatory phase to secrete several cytokines and growth factors (Barrientos et al., 2008). The activated phenotype is marked by changes in the cytoskeleton (i.e., expression of prekeratins K6 and K16) and plasma membrane receptors essential for re-epithelialization, allowing keratinocytes to migrate towards the wound to fill the defect (Coulombe, 1997). For successful wound healing, keratinocytes should be able to not only detach from the underlying basal lamina but also to move and migrate through fibrin and ECM of the wound, a process facilitated by MMP-1, which is expressed at high levels at the wound edges (Pastar et al., 2014) (Figure 1).

Keratinocyte migration from the free edges of the wound takes place within 24 h. As reported in *Keratinocytes, Stem Cells and Epidermal Homeostasis* above, the major contribution to the new cells needed to close the wound is given by ESCs located in stem niches of hair follicle bulges, whereas ESCs of the basal layers of the interfollicular epidermis likely play a minor role (Jones, 1997; Ito et al., 2005). Migration is accompanied by morpho-functional changes of the keratinocytes: in resting phase they appear as cuboid-shaped basal cells reciprocally connected by desmosomes and fixed over their basement membrane by hemidesmosomes. A few hours after wounding, keratinocytes become flattened and elongated, lose their cell-cell and cell-matrix junctions, detach cytoskeletal intermediate filaments from the inner aspect of plasma membrane at the junctional level, show a thick network of contractile filaments in the cortical cytoplasm extending into newly formed lamellipodia, all typical features of EMT occurring during embryo development. While keratinocytes are migrating, their proliferative potential is inhibited. Migrating basal cells are thought to express specific surface markers such as CD44, at variance with resting basal cells (Lü et al., 2013; Pastar et al., 2014; Michopoulou and Rousselle, 2015).

The mechanisms of wound re-epithelialization have not been completely unveiled. The most commonly accepted model is the “leap frog” theory, whereby keratinocytes migrate two or three cell lengths from their initial position and slide or roll over the similar cells previously implanted in the wound (Figure 1). In this way, the epidermal border progressively advances and closes the defect. Such movement depend on surface integrins interacting with fibronectin and newly formed collagen molecules in the wound bed. Keratinocytes are also capable to slide under the scab by exploiting the underlying moist environment (this may also explain the success of occlusive dressings in speeding wound healing). Among the stimuli required for re-epithelialization, TGF-α, keratinocyte growth factor (KGF), and EGF have been identified. Migrating keratinocytes can also produce MMPs to remove damaged matrix: of note, keratinocytes secrete MMP-1/collagenase when in contact with fibrillar collagens of the wound bed, but this secretion is stopped as soon as a new basement membrane is formed and the wound is re-epithelialized. (Steffensen et al., 2001; Li and Kirsner, 2005; Lü et al., 2013; Pastar et al., 2014; Michopoulou and Rousselle, 2015). Taken together, these data suggest that keratinocytes require proper micro-mechanical stimuli to activate their wound closure abilities, which may be altered or absent in low gravity conditions.

During wound healing, keratinocytes can also modulate the functional activity of stromal cells. *In vitro* experiments on skin-equivalent models show that keratinocyte conditioned medium downregulates the production of the profibrotic cytokines TGF-ß and connective tissue growth factor (CTGF) by dermal fibroblasts. Of note, while normal keratinocytes increase fibroblast proliferation but simultaneously reduce collagen production and increase MMP-1 expression and collagen breakdown, thereby promoting normal wound healing, keratinocytes from keloids show higher, and persistent proliferation rates and induce an abnormal, pro-fibrotic phenotype of dermal fibroblasts (Limandjaja et al., 2018).

## Conclusion

The literature on skin wound healing in weightlessness is relatively poor. *In vitro* studies on immune cells, fibroblasts, endothelial, and epithelial cells cultured both in real and modeled micro-gravity conditions show alterations of functions involved in wound healing, such as phagocytosis, adhesion/migration, apoptosis, proliferation, intercellular cross-talking, production of inflammatory mediators, ECM molecules, and growth factors, etc. On the other hand, the studies on animal models in unloading conditions are scanty and insufficient to get definite conclusions. In astronauts, impaired immune function, signs of chronic inflammation, metabolic alterations and skin atrophy have been observed, all factors capable to jeopardize the known skin repair mechanisms (Farahani and DiPietro, 2008; Cialdai and Monici, 2013). The space biology community is aware that only a few wound healing studies have been performed in a real microgravity environment. To fill this gap, specific experiments have been scheduled for being carried out at the ISS in the next months: these have been designed by an international multi-disciplinary research team with the aim to provide further insight into the effects of real unloading conditions on surgically wounded and sutured human skin tissues (Monici et al., 2019; 2021). Despite this uncertain scenario, careful appraisal of the literature suggests a possible general response to microgravity across various cell, tissue, and organ experimental models, consisting of a partial inhibition of the transition of stem cells towards TA cells and terminally differentiated adult cells (Blaber et al., 2014). Thus, gravity-related micro-mechanical stimulation appears a fundamental need for tissues to maintain their regenerative potential and overall health and emerges as a basic feature of mammalian life on Earth under normal gravity load. This notion also suggests that long-lasting microgravity may result in compromised tissue homeostasis and wound healing capability. For this reason, management of wounds during enduring space missions represents a very challenging issue, especially considering the limited availability of diagnostic and therapeutic tools and, likely, the lack of a specialized on-board medical staff. In low-orbit missions, the management of traumatic and surgical emergencies consists of patient stabilization and rapid return to Earth. In future interplanetary missions, timely medical evacuation to Earth would not be possible, nor would telemedicine by either surgical robots or remote guidance of crew medical actions because of communication lag. Nonetheless, the rapid advancements in robotics allow to foresee that the medic/paramedic crew could be assisted by specifically designed robots, programmed to perform basic medical interventions, such as surgical sutures, anaesthesia and vital signs monitoring, as well as diagnostic procedures by ultrasound, computed tomography scan or magnetic resonance imaging, thus improving the capability of on-board assistance to severely ill or injured astronauts (Pantalone et al., 2021). This bio-engineering challenge also underscores the need for further studies on wound healing in space to better understand the problems and define adequate countermeasures.
